# Supplementary material for: FSAP Protects against Histone-Mediated Increase in Endothelial Permeability In Vitro
Source: Int J Mol Sci. 2022 Nov 8;23(22):13706. doi: 10.3390/ijms232213706 (PMC9690979; doi:10.3390/ijms232213706)
Supplement: Supplementary file 1 [file ijms-23-13706-s001.zip › ijms-1966449-supplementary.pdf]

Supplementary Figure S1

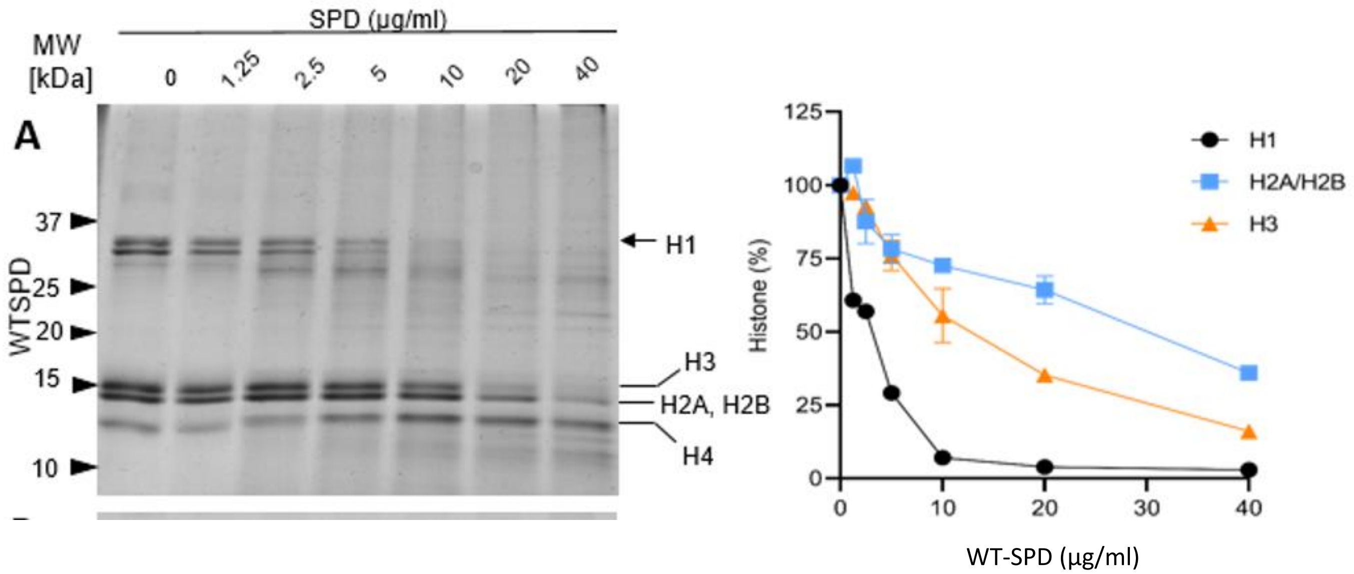

Histone degradation by WT-SPD: Increasing concentrations (0, 1.25, 2.5, 5, 10, 20, and 40 µg/ml) of WT-SPD was incubated with 1 mg/ml histone mix for 1 h at 37°C. The samples were analyzed by running SDS-PAGE followed by Coomassie Staining. Histone H1, H2A/H2B and H3 bands are indicated with arrows. Densiometric analyses of the histone subtypes (% compared to control) is shown in the right panel. Each gel picture is representative of three repeated experiments (n=3). The panel on the right shows mean ± SEM (n=3 experiments). MI-SPD had no effect on degradation of histones (data not shown).

Supplementary Figure S2

Histone H1.0

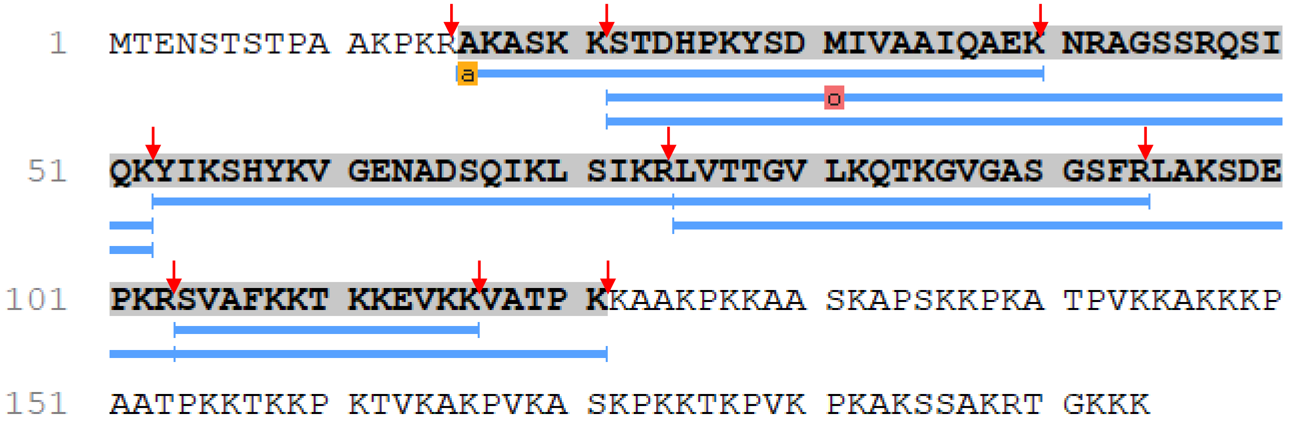

Histone H1.1

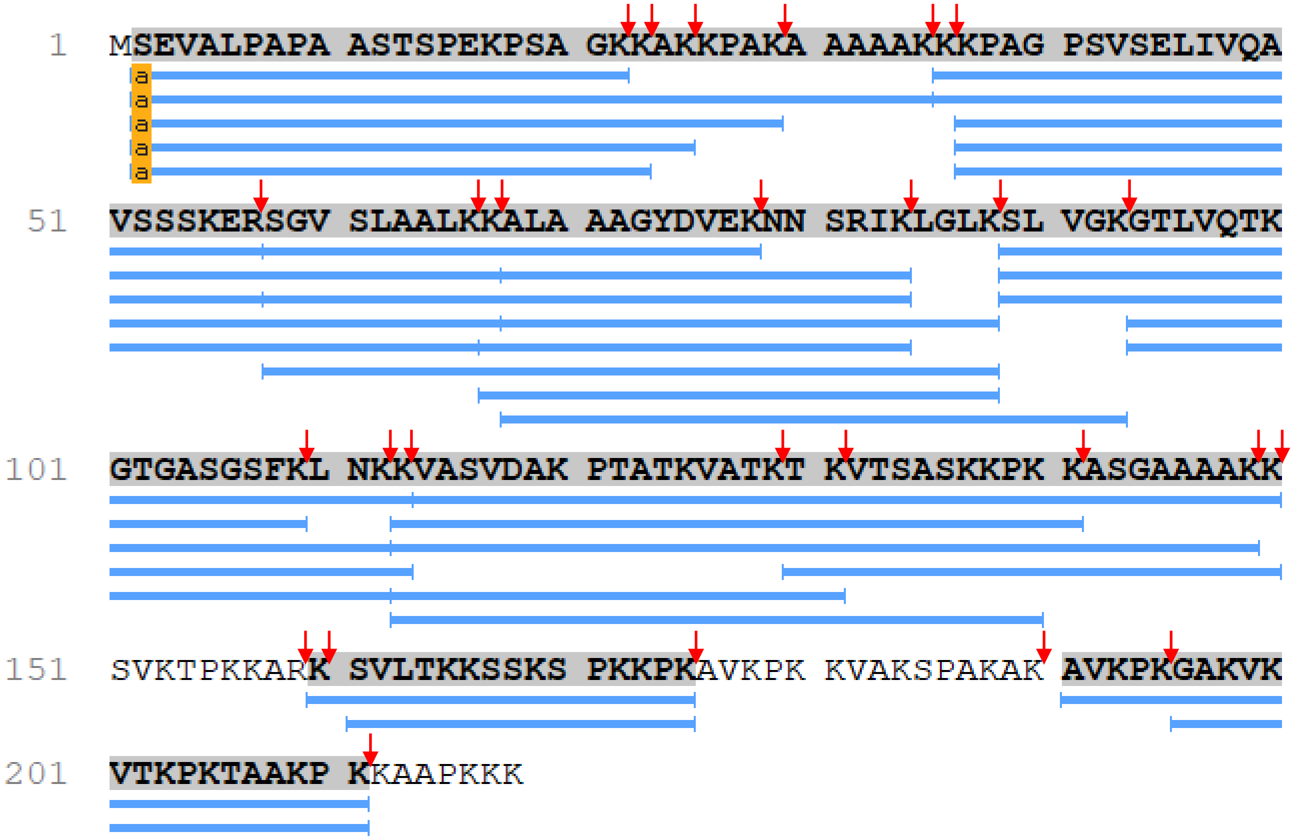

Supplementary Figure S2 continued

Histone H1.2

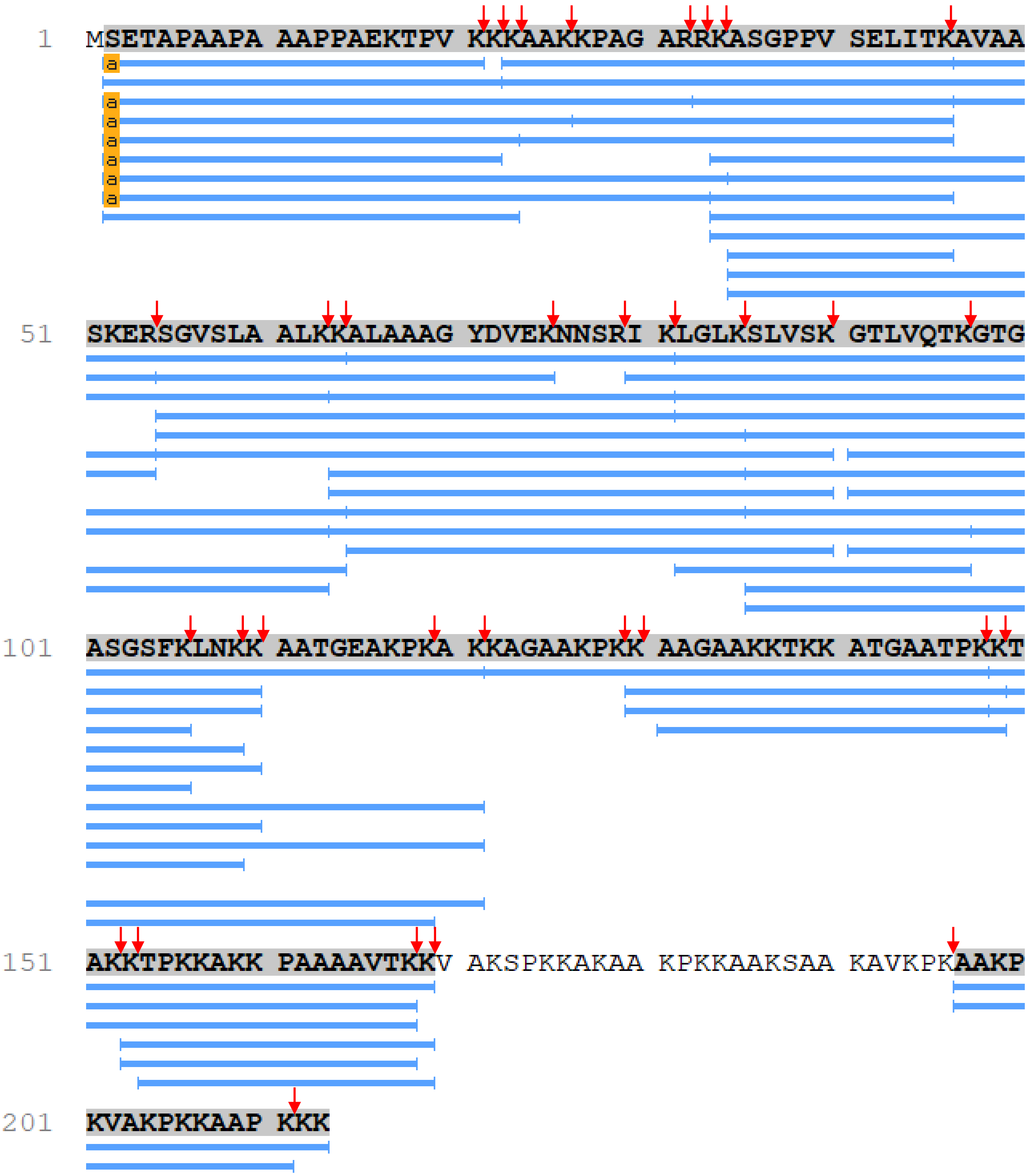

Supplementary Figure S2 continued

Histone H1.3

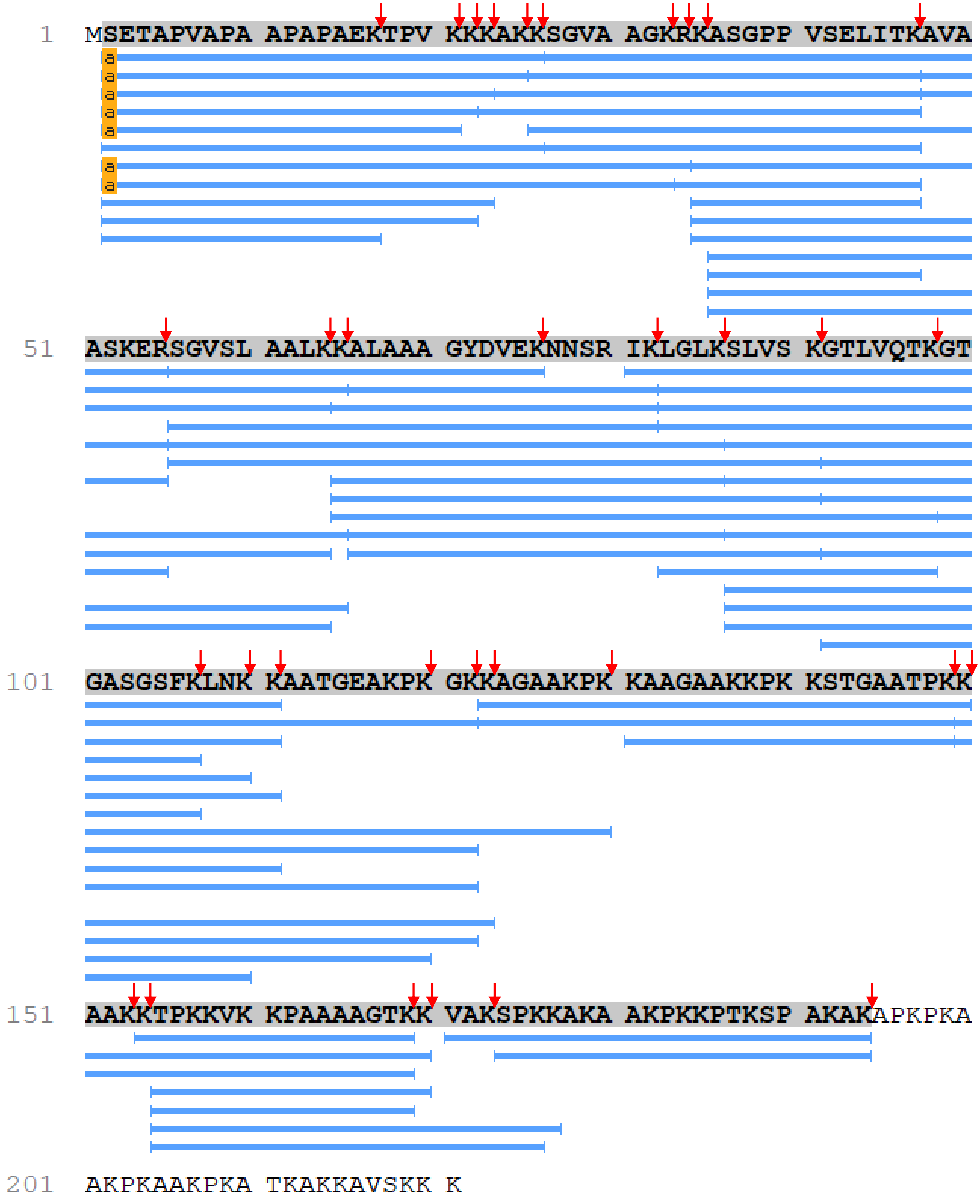

Supplementary Figure S2 continued

Histone H2A1

1 MSGRGKQGGK AR**AKAKTRSS RAGLQFPVGR VHRLLRK**GNY AERVGAGAPV

51 YLAAVLEYLT AEILELAGNA ARDNKKTR**II PRHLQLAIRN DEELNKLLGK**

101 **VTIAQGGVLP NIQAVLLPKK TESHHKAKGK**

Histone H2AJ

1 MSGRGKQGGK VRAKAK**SRSS RAGLQFPVGR VHRLLRK**GNY AERVGAGAPV

51 YLAAVLEYLT AEILELAGNA ARDNKKTR**II PRHLQLAIRN DEELNKLLGK**

101 **VTIAQGGVLP NIQAVLLPK**K TESQKTKSK

Supplementary Figure S2 continued

Histone H2AV

1 MAGGKAGKDS GKAKAKAVSR SQRAGLQFPV GRIHRHLKTR TTSHGRVGAT

51 AAVYSAAILE YLTAEVLELA GNASKDLKVK RITPRHLQLA IRGDEELDSL

101 IKATIAGGGV IPHIHKSLIG KKGQQKTA

Histone H2AZ

1 MAGGKAGKDS GKAKTKAVSR SQRAGLQFPV GRIHRHLKSR TTSHGRVGAT

51 AAVYSAAILE YLTAEVLELA GNASKDLKVK RITPRHLQLA IRGDEELDSL

101 IKATIAGGGV IPHIHKSLIG KKGQQKTV

Supplementary Figure S2 continued

Histone H2B1

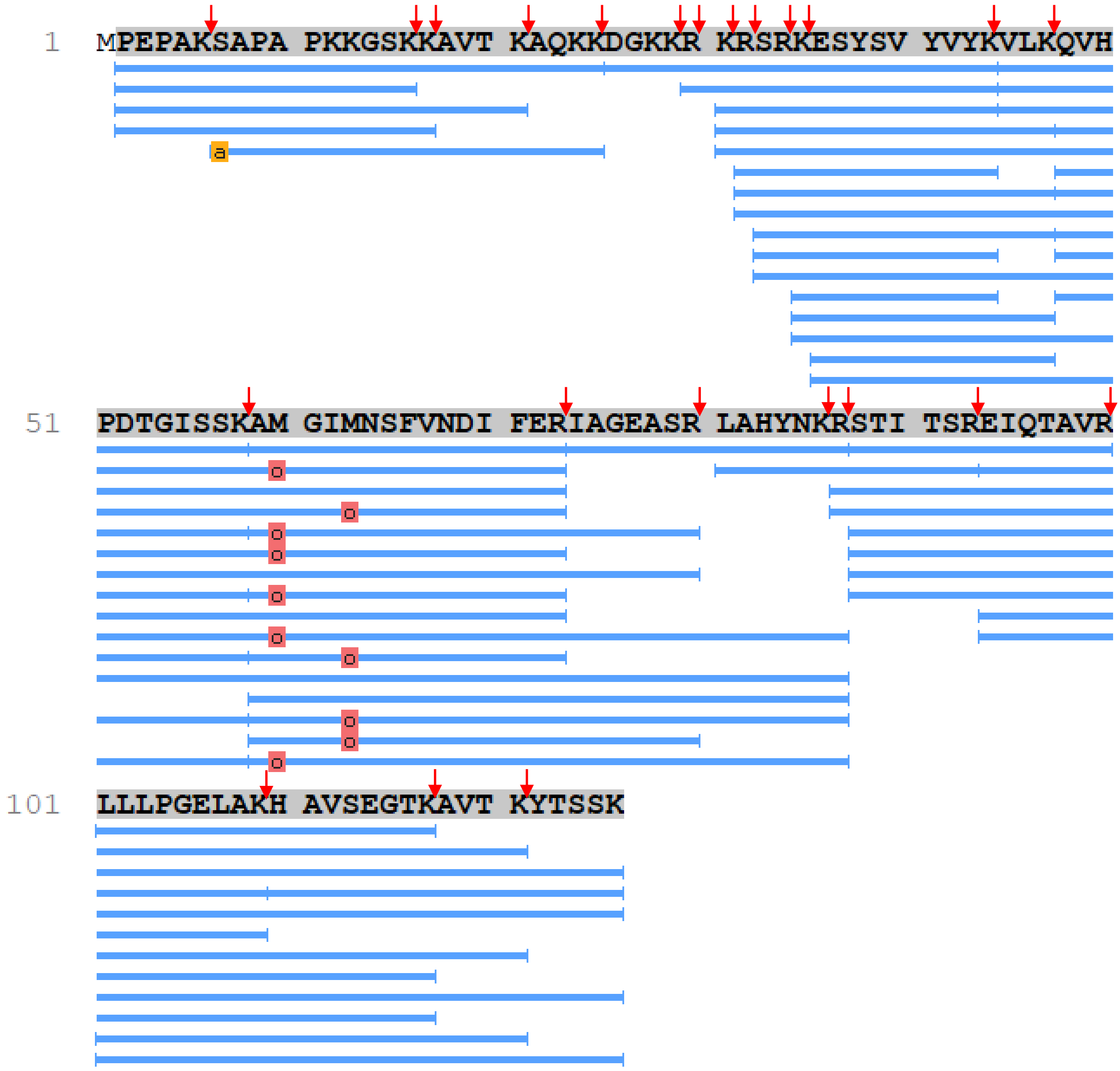

Supplementary Figure S2 continued

Histone H3.1/H3.2

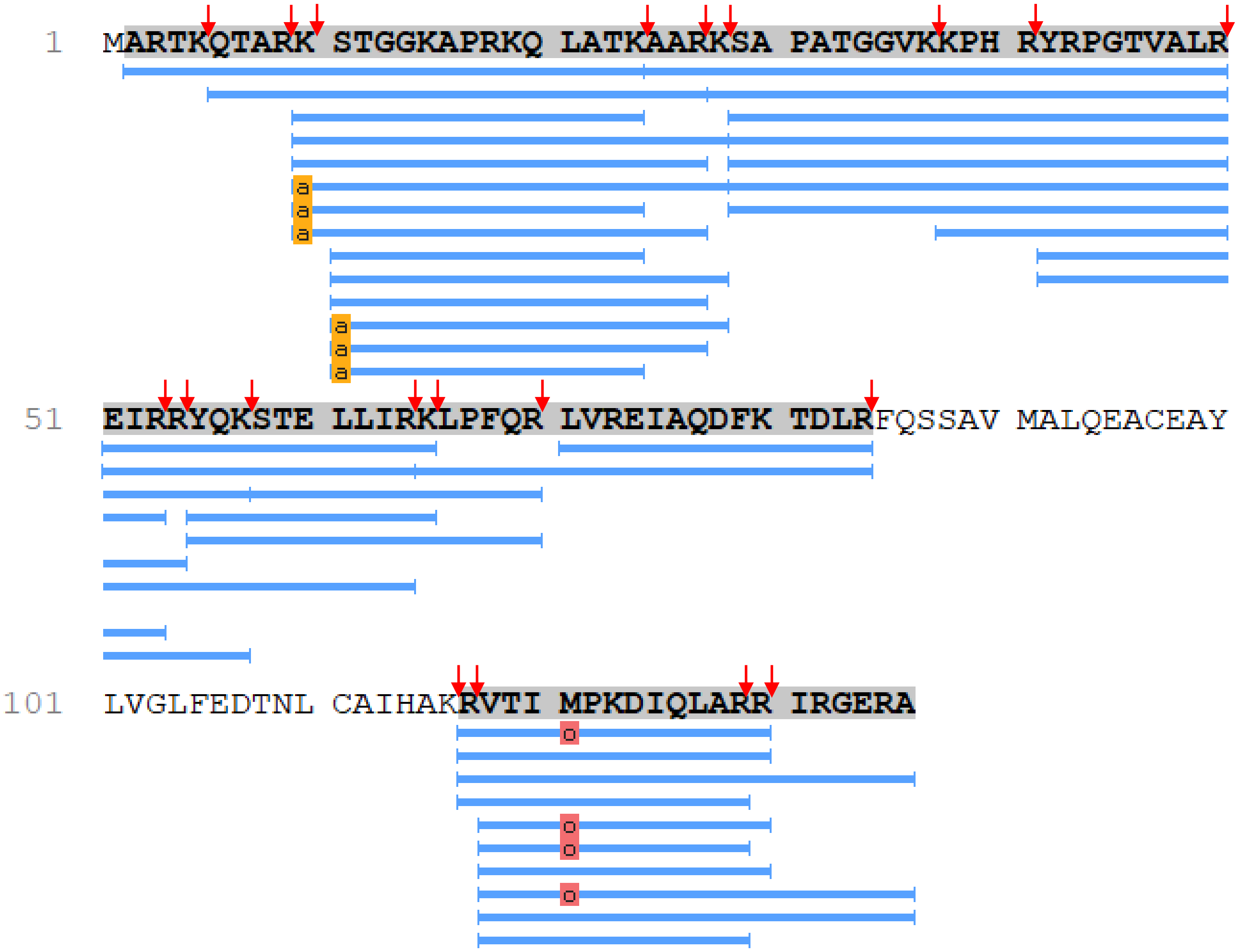

Supplementary Figure S2 continued

Histone H3.3

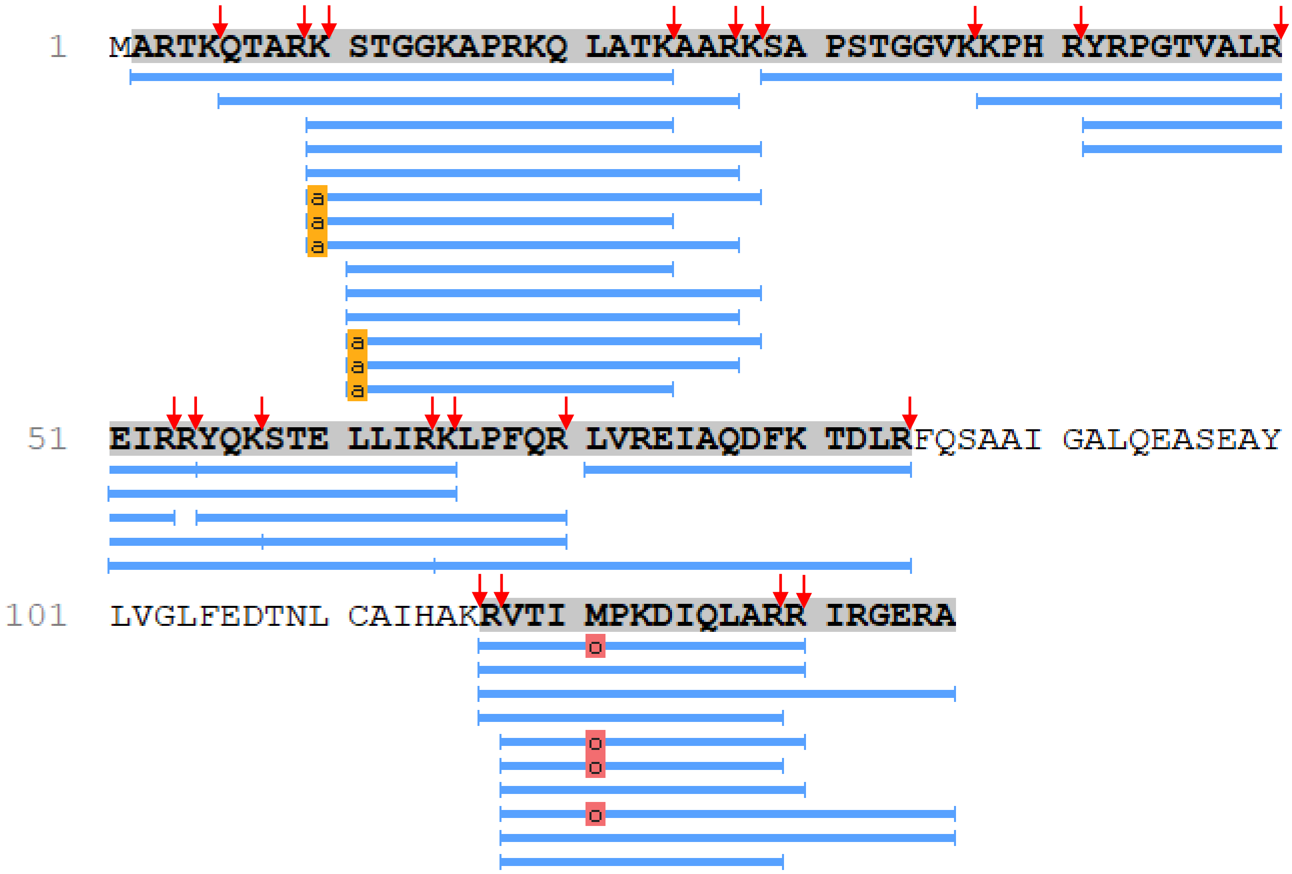

Supplementary Figure S2 continued

Histone H4

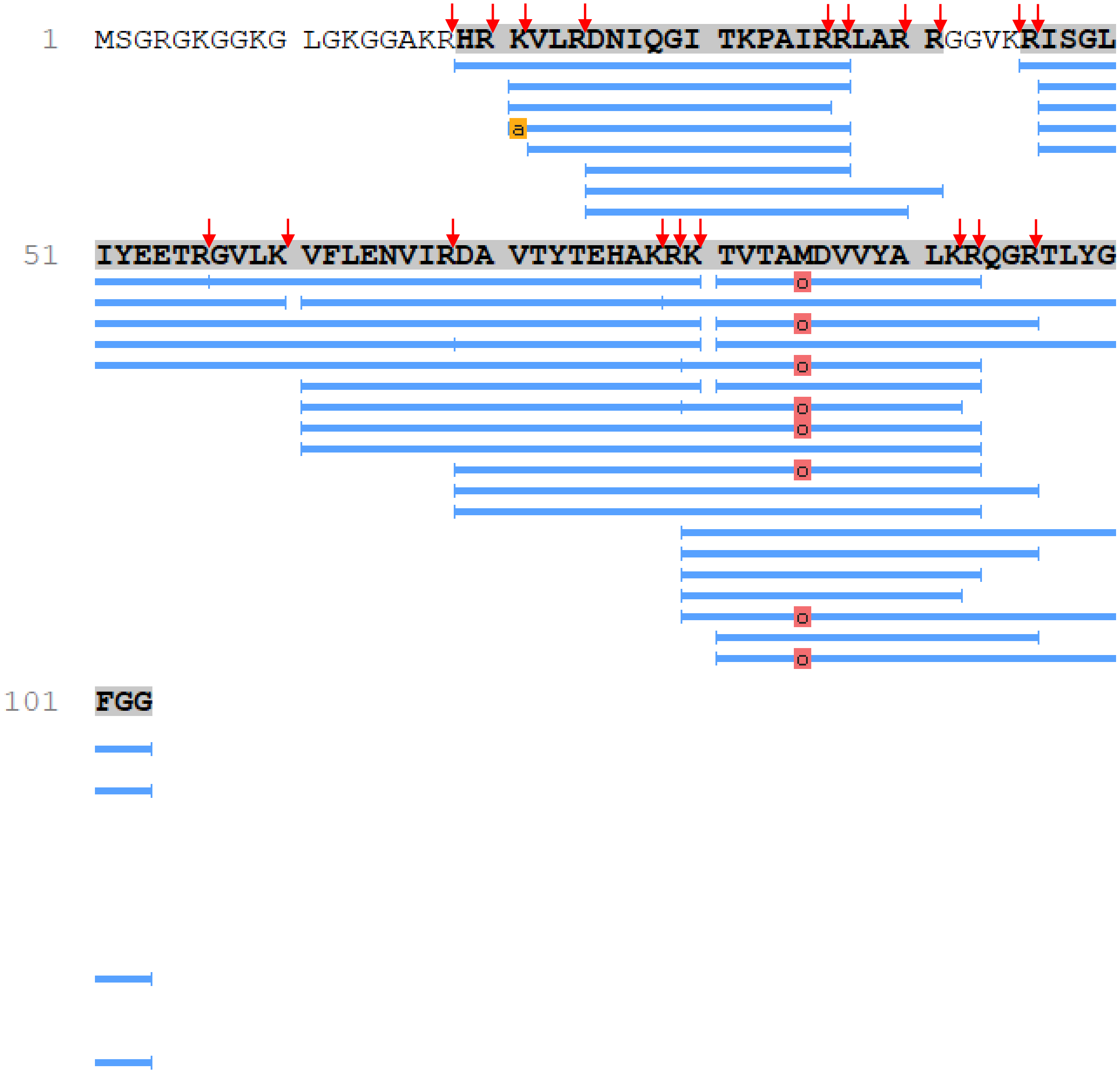

Identification of WT-SPD cleavage sites in histones by mass spectrometry: In order to identify the histone composition of the histone mix as well as the cleavage sites in histones, samples of histones were incubated with WT-SPD and analyzed by LC-MS (Bruker Daltonik, Bremen, Germany) using a nanoElute nanoflow liquid chromatography system coupled to a TimsTOF Pro via a CaptiveSpray nanoelectrospray ion source (Bruker Daltonik, Bremen, Germany). The peptides were separated on a reversed phase C18 column (25 cm x 75 µm, 1.6 µm, IonOpticks (Fitzroy, VIC, Australia) at 50°C. Mobile phase A contained water with 0.1% (vol/vol) formic acid, and acetonitrile with 0.1% (vol/vol) formic acid was used as mobile phase B. The peptides were separated by a gradient from 0-35% mobile phase B over 54 min at a flow rate of 300 nl/min at a column temperature of 50°C. MS acquisition was performed in DDA-PASEF mode. The LC/MS data were searched against bovine histone sequences from UniProt, with PEAKS X+ software version 10.5 (Bioinformatics Solutions, Waterloo, ON, Canada).
